# Using “Functional” Target Coordinates of the Subthalamic Nucleus to Assess the Indirect and Direct Methods of the Preoperative Planning: Do the Anatomical and Functional Targets Coincide?

**DOI:** 10.3390/brainsci6040065

**Published:** 2016-12-21

**Authors:** Ahmed Rabie, Leo Verhagen Metman, Konstantin V. Slavin

**Affiliations:** 1 Department of Neurosurgery, University of Illinois at Chicago, Chicago, IL 60612, USA; dr_a_rabie@hotmail.com; 2 Department of Neurosurgery, Alexandria University, Alexandria, Egypt; 3 Department of Neurological Sciences, Rush University Medical Center, Chicago, IL 60612, USA; Leonard_A_Verhagen@rush.edu

**Keywords:** subthalamic nucleus, deep brain stimulation, targeting, Parkinson’s disease, planning

## Abstract

Objective: To answer the question of whether the anatomical center of the subthalamic nucleus (STN), as calculated indirectly from stereotactic atlases or by direct visualization on magnetic resonance imaging (MRI), corresponds to the best functional target. Since the neighboring red nucleus (RN) is well visualized on MRI, we studied the relationships of the final target to its different borders. Methods: We analyzed the data of 23 PD patients (46 targets) who underwent bilateral frame-based STN deep brain stimulation (DBS) procedure with microelectrode recording guidance. We calculated coordinates of the active contact on DBS electrode on postoperative MRI, which we referred to as the final “functional/optimal” target. The coordinates calculated by the atlas-based “indirect” and “direct” methods, as well as the coordinates of the different RN borders were compared to these final coordinates. Results: The mean ± SD of the final target coordinates was 11.7 ± 1.5 mm lateral (X), 2.4 ± 1.5 mm posterior (Y), and 6.1 ± 1.7 mm inferior to the mid-commissural point (Z). No significant differences were found between the “indirect” X, Z coordinates and those of the final targets. The “indirect” Y coordinate was significantly posterior to Y of the final target, with mean difference of 0.6 mm (*p* = 0.014). No significant differences were found between the “direct” X, Y, and Z coordinates and those of the final targets. Conclusions: The functional STN target is located in direct proximity to its anatomical center. During preoperative targeting, we recommend using the “direct” method, and taking into consideration the relationships of the final target to the mid-commissural point (MCP) and the different RN borders.

## 1. Introduction

Deep brain stimulation (DBS) is the gold standard surgical treatment of advanced Parkinson’s disease (PD). The subthalamic nucleus (STN) has been used for the last two decades as the target of choice for this procedure [1].

The STN is a small gray matter structure located at the junction of the midbrain and diencephalon. It has anatomic relationships to the internal capsule and the Globus Pallidus Internus (GPi) anterolaterally, the Zona Incerta (ZI) and the thalamus superiorly, fibers of the third nerve anteromedially, the red nucleus (RN) posteromedially, and the cerebral peduncle and the Substantia Nigra (SN) ventrally [2,3]. The target of DBS is the sensorimotor (dorsolateral) part of the STN [4,5]. This complex anatomy of the STN necessitates precise targeting during DBS surgeries.

There are two conventional methods for pre-operative localization of the STN [6]. The first is the indirect targeting; in which a brain atlas is used to define the coordinates of the STN in relation to the midcommissural point (MCP). The second is the direct method, which was developed by the work of Bejjani et al. in 2000 [7] in which the STN is directly visualized as a hypointense structure on T2 weighted images. Direct localization became easier with the improvements of magnetic resonance imaging (MRI) sequences and the use of special stereotactic software to perform 3D image reconstruction and help in the calculations [8,9,10,11,12]. Many studies assessed the direct coordinates and compared them to the coordinates obtained by the indirect method [13,14,15,16,17]. Yet, the exact correlation between these two conventional methods and the postoperative final (functional) target has not been established.

In this paper, we considered the final position of the best active electrode contact on the postoperative MRI images as the “true functional stimulation site” or the “final target”. This final target position is confirmed by the intra-operative microelectrode recording (MER) and the postoperative improvement of the Parkinsonian symptoms. We compared these final coordinates to the initial coordinates calculated by both the direct and indirect methods.

## 2. Objectives

Assessment of the accuracy of the conventional methods of “direct” and “indirect” localization of STN target vs. the final functional target. This may help us to determine the optimal coordinates for the STN DBS target. We also aim to study the relationships of the final functional target coordinates to the coordinates of the different borders of the RN, in attempt to find any new relationships that can improve the preoperative planning. This may eventually increase the accuracy of the preoperative targeting, and subsequently decrease the intra-operative time needed for the MER and the number of microelectrode insertion tracks needed to reach the target, and consequently reducing the rate of complications.

## 3. Methods

After obtaining an appropriate institutional review board (IRB) approval, we retrospectively analyzed the data of all patients diagnosed with advanced Parkinson’s disease who underwent bilateral STN-DBS at the University of Illinois at Chicago (UIC) in the period from January 2013 to December 2014. From a total of 40 bilateral STN-DBS procedures that were performed over this period, we included in this analysis 23 patients (46 targets) who had available postoperative MRI images, and a minimum follow up period of 6 months with documented clinical improvement on fixed stimulation parameters. We excluded the patients with postoperative complications causing abnormalities in the electrode position (1 patient), those who did not get a beneficial clinical effect from the stimulation (1 patient), and those whose follow-up visits (15 patients) took place in other institutions.

### 3.1. The Preoperative Planning and the Surgical Procedure

The surgery was done in two stages. The first stage involves implantation of the DBS electrodes under local anesthesia. Procedure starts with application of Leksell stereotactic frame Model G (Elekta Instruments, Atlanta, GA, USA) to the patient’s head.

**Figure 1:**
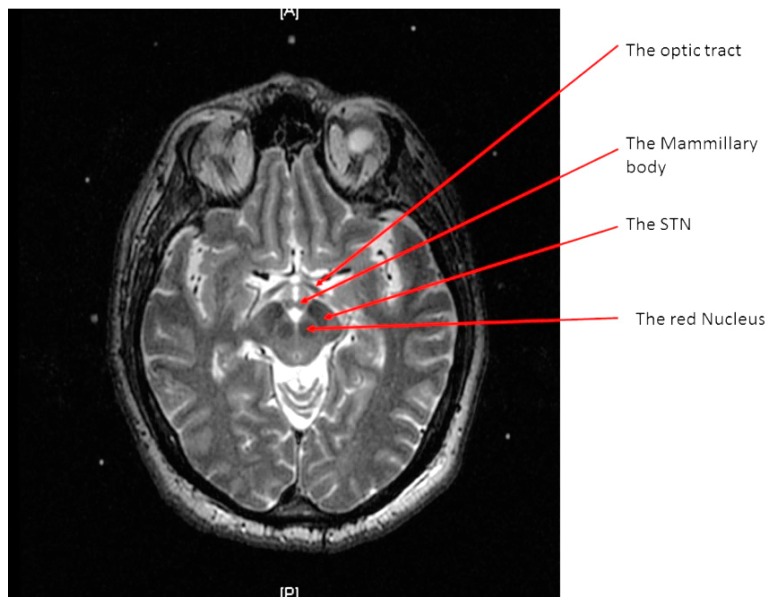
An axial T2 weighted magnetic resonance image (MRI) at the level of the midbrain showing the two subthalamic nuclei (STN).

A high-resolution MRI of the patient’s brain with a 3 Tesla scanner (Signa 3T94 VHi; General Electric Medical Systems, Milwaukee, WI, USA) was done. Two main sequences were obtained. The first is a 3D T1-weighted, spoiled gradient echo imaging of the entire head (section thickness: 2 mm; field of view: 26 × 26 cm; TR: 7.0–8.0 milliseconds; TE: ~400 milliseconds; flip angle: 12; band width: 31.25 KHz; acquisition time: <7 min). The second is high-resolution, contiguous, T2-weighted, fast spin-echo imaging through the region of the midbrain and basal ganglia (section thickness: 1.5 mm; slice interval: 0 mm; matrix size: 512 × 512; field of view: 26 × 26 cm; TR: 4600–6200 milliseconds; TE: 95–108 milliseconds; acquisition time: <5 min) (Figure 1).

#### 3.1.1. The “Indirect” Method of the STN Coordinates Calculation

**Figure 2:**
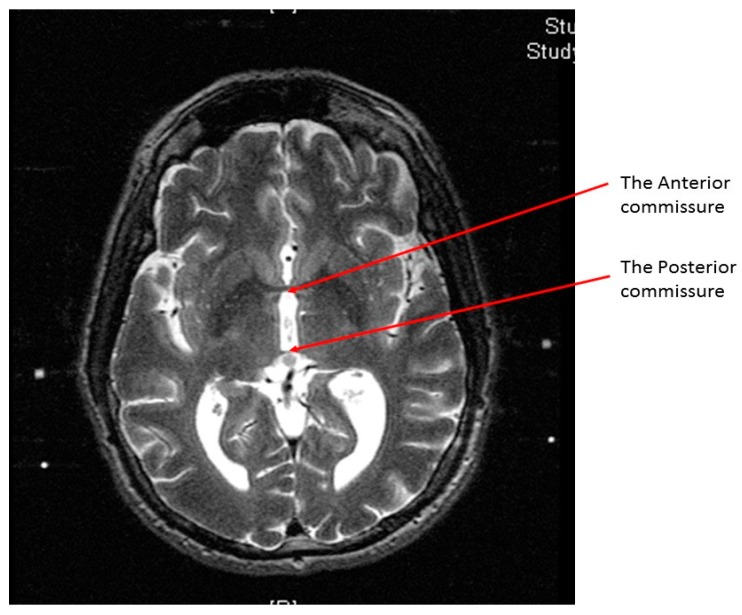
An axial T2 weighted magnetic resonance image showing the anterior commissure and the posterior commissure at the same cut.

**Figure 3:**
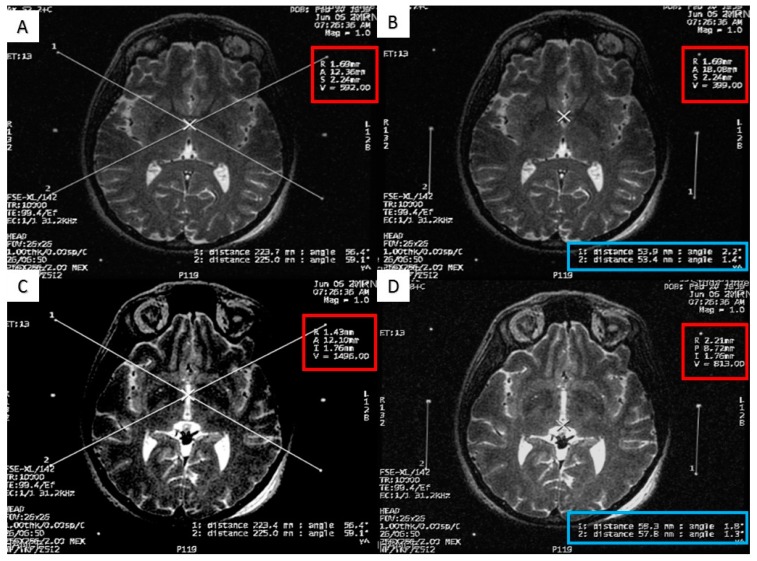
Calculating the anterior commissure (AC) and posterior commissure (PC) coordinates using the magnetic resonance scanner console. (**A**) Two diagonal lines intersecting at the center of the frame at the AC level with the magnetic resonance imaging (MRI) coordinates of the center of the frame shown inside the red square; (**B**) a crosshair at the posterior margin of the AC, with the MRI coordinates of the AC shown inside the red square. Two lines are drawn between the middle and the lower fiducials on both sides of the frame and their lengths (in the blue rectangle) are used to calculate the Z coordinate of the AC; (**C**) two diagonal lines intersecting at the center of the frame at the PC level with the MRI coordinates of the center of the frame shown inside the red square; (**D**) a crosshair at the anterior margin of the PC, with the MRI coordinates of the PC shown inside the red square. Two lines are drawn between the middle and the lower fiducials on both sides of the frame and their lengths (in the blue rectangle) are used to calculate the Z coordinates of the PC.

At the end of the scan, we chose the axial T2 image (or two adjacent images) in which the anterior commissure (AC) and the posterior commissure (PC) are identified (Figure 2). Then, we measured the distance between the middle and lower fiducials on both sides of the frame, and a maximum of 2 mm difference was allowed. The X and Y MR coordinate of the center of the frame was obtained at the point of meeting of two diagonal lines drawn on the MR console between the opposing anterior and posterior fiducials. After that, the X and Y MR coordinates of both the AC, the PC, and the center of the frame (Figure 3) were obtained from the MR console, and entered into a simple Excel worksheet (Microsoft, Redmond, WA, USA) designed by the senior author.

Afterwards, the frame coordinates of the AC, PC, the mid-commissural point (MCP), and the intercommissural distance (should be from 21 to 28 mm) were calculated with the help of this Excel worksheet by using the following formulas:

X coordinates of the AC = 100 + MRI scanner X coordinates of the AC − MRI scanner X coordinates of the center of the frame
(1)

Y coordinates of the AC = 100 + MRI scanner Y coordinates of the AC − MRI scanner Y coordinates of the center of the frame
(2)

Z coordinates of the AC = 40 + distance between the lower and middle fiducials at the AC-PC plane
(3)

Intercommisural distance = √ [(X_AC_ − X_PC_) × (X_AC_ − X_PC_) + (Y_AC_ − Y_PC_) × (Y_AC_ − Y_PC_) + (Z_AC_ − Z_PC_) × (Z_AC_ − Z_PC_)] (4)

X_MCP_ = (X_AC_ + X_PC_)/2 (5)

Y_MCP_ = (Y_AC_ + Y_PC_)/2 (6)

Z_MCP_ = (Z_AC_ + Z_PC_)/2 (7)

Based on the known anatomical relationship of the STN to the MCP from previous anatomical studies and stereotactic atlases [18,19,20,21], we selected the STN target at 12 mm lateral, 3 mm posterior, and 6 mm inferior to the MCP. We used the following formulas to do the calculations:

X_STN_ = X_MCP_**± 12 mm** (8)

Subtract for the right STN and add for the left STN

Y_STN_ = Y_MCP_**− 3 mm** (9)

Z_STN_ = Z_MCP_**+ 6 mm** (10)

The base of the STN.

#### 3.1.2. The “Direct” Method of the STN Coordinates Calculation

**Figure 4:**
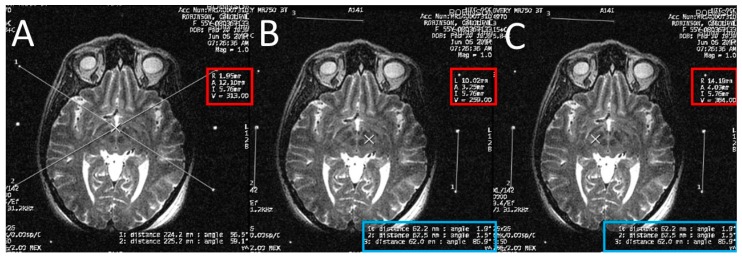
Calculating the subthalamic nucleus (STN) coordinates from the magnetic resonance imaging (MRI) console. (**A**) two diagonal lines intersecting at the center of the frame at the STN level with MRI coordinates of the center of the frame shown inside the red square; (**B**) a crosshair at the center of the left STN, with its MRI coordinates shown inside the red square, two line are drawn between the middle and lower fiducials on both sides of the frame and their lengths (in the blue rectangle) are used to calculate the Z coordinate; (**C**) a crosshair at the center of the right STN, with its MRI coordinates shown inside the red square, two line are drawn between the middle and lower fiducials on both sides of the frame and their lengths (in the blue rectangle) are used to calculate the Z coordinate.

The STN is the hypointense structure located lateral and anterior to the red nucleus on axial T2 MRI (Figure 4) [8]. The center of the STN hypointensity was identified at the extension of a straight line drawn at the anterior margin of the RN bisecting the STN. Then, the coordinates were calculated using the same Excel worksheet.

**Figure 5:**
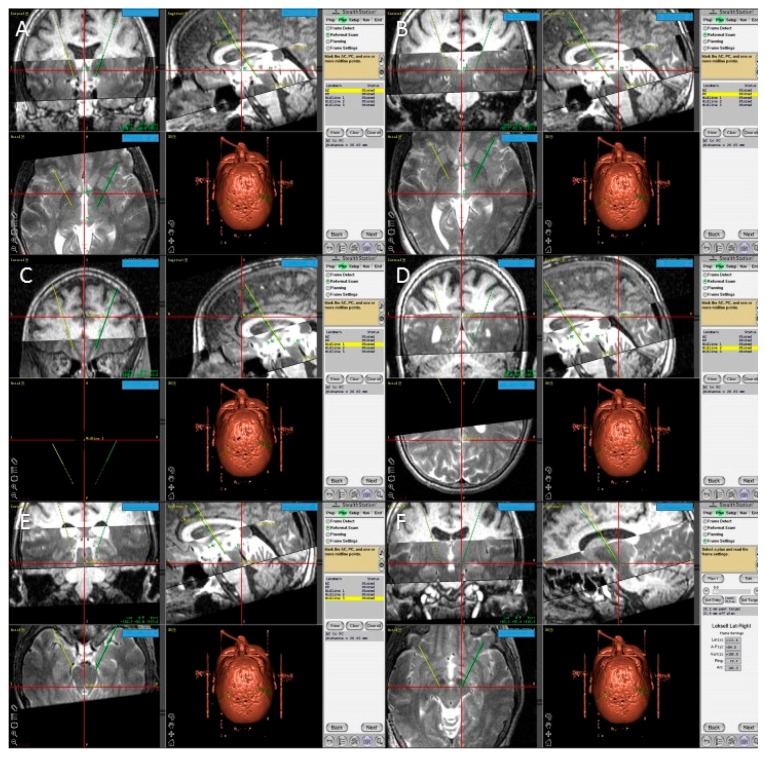
Screen shots from the Framelink software of the Stealthstation showing fused T1 and T2 MRI images of the patient and the planning process with identification of the posterior edge of the anterior commissure (AC) (**A**); the anterior edge of the posterior commissure (PC) (**B**); three midline points (**C**–**E**); and the final coordinates of the right subthalamic nucleus (**F**).

In the operation room, we used the FrameLink software, which is a part of StealthStation navigation system (Medtronic, Minneapolis, MN, USA) to confirm our calculations of the direct STN coordinates (Figure 5). This software compensates for head and frame tilt in any direction. The final coordinates for the procedure were derived from the two techniques, and subsequently adjusted using intraoperative electrical microrecording and macrostimulation.

**Figure 6:**
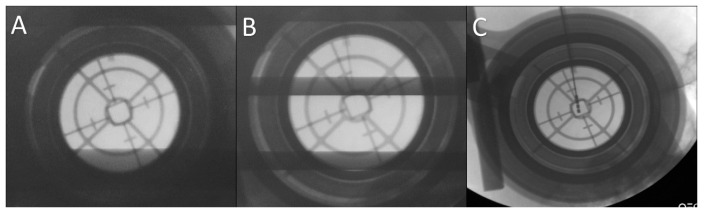
Fluoroscopic confirmation of the target approach. (**A**) Confirmation of the position of the stereotactic cannula; (**B**) the microelectrode is advanced to the target under fluoroscopic guidance; (**C**) the final position of the deep brain stimulation (DBS) electrode confirmed.

During surgery, we performed microelectrode recording (MER) of the brain activity using a NeuroNav microelectrode recording system (AlphaOmega, Nazareth, Israel). Fluoroscopic confirmation of the target approach was obtained at 5 mm intervals, 2 mm above the target, and at the target (Figure 6).

**Figure 7:**
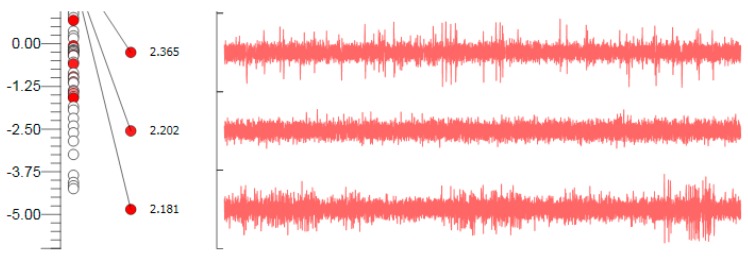
Microelectrode recording appearance of the subthalamic nucleus signal; note the increase in background activity, with high amplitude irregular firing.

During MER, the STN is the most electrically active structure encountered during the recording. An indicator of entry into the STN is the increase of the background activity (Figure 7). The STN cells have a mean firing rate of 37 ± 17 Hz with high amplitude and irregular firing pattern [22]. We used the following criteria for choosing an ideal STN target:

- The length of the STN, measured along its trajectory, is 4–5 mm.
- Dense discharge patterns recorded in the STN.
- The presence of an identifiable region of increased neuronal firing at the STN on sensorimotor stimulation of the contralateral limbs.

After identification of the STN borders and depth by the MER, we started high frequency macrostimulation. The aim of the stimulation was to confirm the optimal target, which provides adequate control of the parkinsonian symptoms (most identifiable is the tremor), with no undesirable effects from stimulation below 4 V.

**Figure 8:**
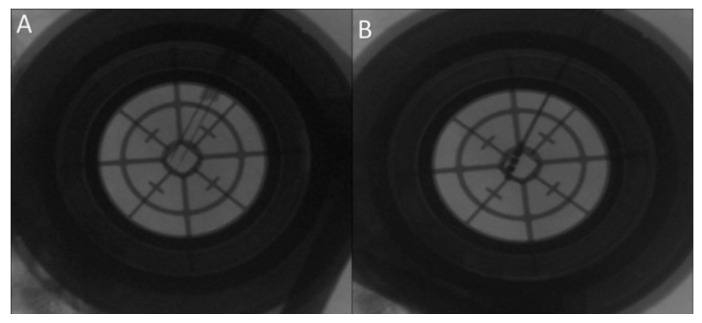
Fluoroscopic confirmation of the electrode position showing (**A**) a second microelectrode is inserted posterior to the original one due to suboptimal subthalamic nucleus (STN) signal during mapping along the original trajectory; (**B**) the final position of the deep brain stimulation (DBS) electrode in the new posterior trajectory.

We tried to minimize the number of the tracks used for recording and stimulation to reach the STN as possible (Figure 6). The maximum number of tracks we used for a single side target was three (Figure 8).

Once we reached our desired target, we removed the microelectrode and replaced it with a standard four contact (0–3) deep brain stimulation electrode (Medtronic DBS lead 3389). Generally, we placed the deepest electrode contact (0) at or just beyond the target point. Then, we repeated the testing using this electrode in order to confirm the reproducibility of the beneficial effects and high thresholds for the undesirable effects (Figure 6 and Figure 8). We locked the electrode in place using a Stimloc device (Medtronic, Minneapolis, MN, USA). Then, the same procedure was repeated again for the opposite side electrode. 

After removal of the stereotactic frame, the patient was transferred to the intensive care unit. Upon arrival, all patients had a CT scan of the head to rule out the presence of intracerebral hemorrhage. They all had an MRI of the brain in the same day of surgery or the next day to check the position of the electrodes prior to discharge home.

The patient returned to hospital after one week for the second stage, in which the implantable pulse generator (IPG) was implanted under general anesthesia.

### 3.2. Postoperative Calculation of the Active Contact Coordinates

Postoperative MRI images of the patients were loaded to the Medtronic Stealth station and the Framelink stereotactic software was used to perform fusion of the pre and the postoperative MRI images. This allowed us to get the coordinates of the active DBS electrode in relation to the mid commissural point (MCP). The first step after image fusion was to identify the tip of the DBS electrode. We chose a point at the center of the hypointense signal representing the tip of the electrode in all the three orthogonal planes and we marked it as our target. Then, we identified and marked the entry point of the electrode into the brain. The computer software then was able to draw a trajectory overlapping the electrode’s pathway through the brain. Then, using a probe eye view we could move along this trajectory from the distal tip upwards. We moved by 0.25 mm increments until we reached the predetermined position of the active contact and we got its coordinates in relation to the MCP coordinates.

**Figure 9:**
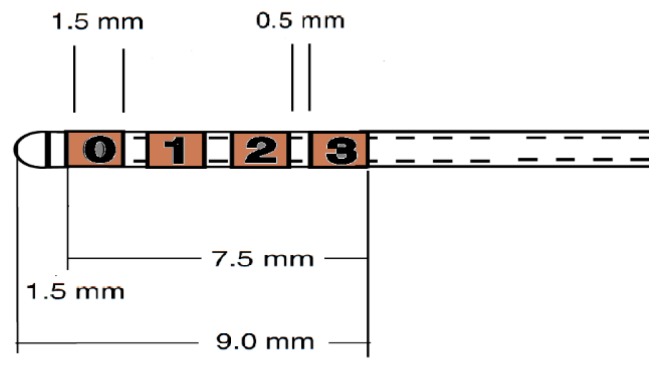
An illustration showing the geometry of the distal end of the 3389 deep brain stimulation (DBS) electrode model (Medtronic, Minneapolis, MN, USA).

As all our patients were followed up for at least a period of 6 months, in our study, we collected the data of the stimulating electrodes combinations that gave them optimal clinical response and no undesirable effects at the lowest stimulation voltage. The Medtronic 3389 electrode which we used has four contacts that can be named 0, 1, 2, 3 (or 4, 5, 6, 7 or 8, 9, 10, 11) with contacts 0, 4 or 8 being the most distal and they are located 1.5 mm proximal to the tip of the electrode (Figure 9). The contacts are 1.5 mm in length and are separated by 0.5 mm intervals. We used a previously published methodology to calculate the coordinates of the active contact [23,24]. The midpoint of the first contact (0, 4, or 8) is located 2.25 mm cranial to the tip of the electrode, the midpoints of the second contact (1, 5, or 9) is located 4.25 mm cranial to the tip, the midpoint of the third contact (2, 6, or 10) is located 6.25 mm cranial to the tip, and the midpoint of the fourth contact (3, 7, or 11) is located 8.25 mm cranial to the tip. If the patient had a double monopolar electrodes combination, we chose our target to be the midpoint between the two cathodes. If he had a bipolar combination, we chose the cathode as our target [23,24].

We also calculated X coordinate of the lateral RN border, Y coordinate of the anterior RN border, and Z coordinate of the superior RN border to compare them with X, Y, and Z coordinates of the active contact respectively.

### 3.3. Data Processing and Statistical Analysis

The final active contact coordinates, being confirmed intra-operatively in all the patients as the STN functional target, and with documented postoperative beneficial clinical effect, were compared to the coordinates obtained by the preoperative indirect atlas based calculations and to those obtained by direct visualization. All coordinates were recorded based on the relationships of the target to the MCP, and all the distances were measured in millimeters. Data were initially recorded using a Microsoft Excel work sheet. We subtracted X, Y, and Z of the final active electrode coordinates from the corresponding X, Y, and Z of direct and indirect STN coordinates. We also calculated the distances between X coordinate of the lateral RN border, Y coordinate of the anterior RN border, and Z coordinate of the superior RN border and the final coordinates. We also calculated the Euclidean distances between the final active contact coordinates and the preoperative direct and indirect targets coordinates in three dimensions. The Euclidean distance is the “ordinary” (i.e., straight-line) distance between two points (*p* and *q*) in the Euclidean space. With this distance, the Euclidean space becomes a metric space. In a three-dimensional system with *p* at (*p*_1_, *p*_2_, *p*_3_) and *q* at (*q*_1_, *q*_2_, *q*_3_). The Euclidean distance is calculated by using the formula [*d*(*p*,*q*) = √ (*p*_1_ − *q*_1_)^2^ + (*p*_2_ − *q*_3_)^2^ + (*p*_3_ − *q*_3_)^2^].

We coded, tabulated, and statistically analyzed our data using the IBM SPSS statistics software version 22.0 (IBM Corp., Chicago, IL, USA). Descriptive statistics were done for quantitative data as minimum & maximum of the range, mean ± SD (standard deviation), median, and confidence interval (CI) while we calculated numbers and percentages for qualitative data, as well as well as 95% confidence interval for both. Inferential analyses were done using the one-sample *t*-test and the paired *t*-test. The null hypothesis was that there is no difference between the direct, the indirect STN targets, the borders of RN, and the final electrode coordinates. The level of significance was taken at *p*-value < 0.05.

## 4. Results

**Figure 10:**
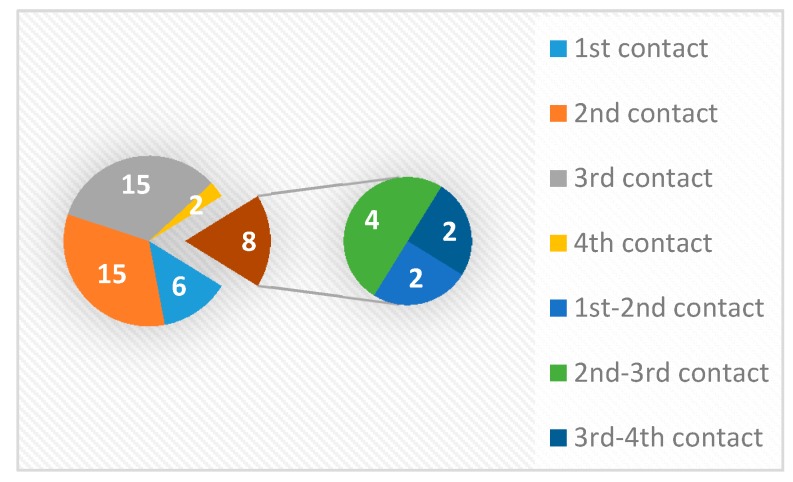
The distribution of the active contacts used as the final targets.

The most commonly used electrode contacts for stimulation were the second (1, 5, 9) and the third contacts (2, 6, 10), and each of them was used in used in 15 targets (32.6%). The most commonly used double monopolar combination was between the second and the third contact (*N* = 4, 8.7%) (Figure 10).

### 4.1. X Coordinates

**Figure 11:**
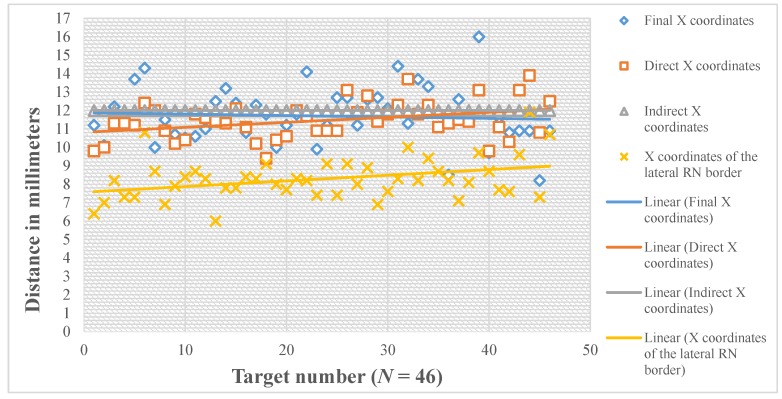
The X coordinates in relation to the mid-commissural point (MCP).

The mean value of X coordinate of the final active contact was 11.7 mm lateral to MCP, with SD of 1.5 mm, median of 11.5, range 8.2–16.0 mm, and 95% CI of 11.2–12.2 mm (Figure 11). Comparison of the direct X coordinate to the final X coordinate showed no statistically significant difference with the mean value of the difference (X direct-X final) is −0.2 mm (95% CI −0.7–0.2 mm) (Table 1). The number of the direct X coordinates that lie within 1 mm lateral and 1 mm medial to the final X coordinates was 24/46 (52.2%, 95% CI = 37.2%–67.2%) (Table 2). Comparison of the indirect X coordinate to the final X coordinate also showed no statistically significant difference with the mean value of the difference (X indirect-X final) was 0.3 mm (95% CI = −0.2–0.8 mm). The number of the indirect X coordinates that lie within 1 mm lateral and 1 mm medial to the final X coordinates was 23/46 (50.0%, 95% CI = 35.0%–65.0%) (Table 2).

The mean value of X coordinate of the lateral RN border was 8.3 mm lateral to MCP (SD of 1.1 mm, median of 8.2, range 6.0–11.9 mm). The mean distance between X coordinates of the lateral RN border and X coordinate of the final target (X of the RN-X final) was 3.4 mm on the medial side (95% CI −4.0–−2.9 mm, *p* < 0.001) (Table 3).

### 4.2. Y Coordinates

**Figure 12:**
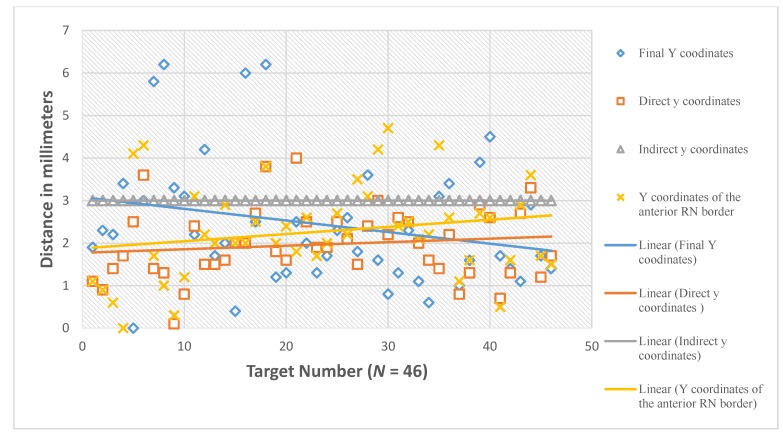
The Y coordinates in relation to the mid-commissural point (MCP).

**Figure 13:**
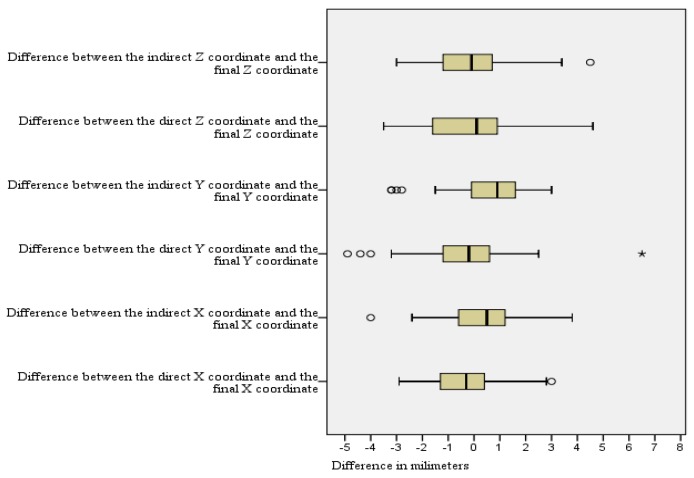
The differences between the direct and indirect coordinates of the subthalamic nucleus from the final active electrode coordinates. **°**: outliers.

The mean value of Y coordinate of the final active contact is 2.4 mm posterior to MCP (SD of 1.5 mm, median of 2.1, range 0–6.2 mm, 95% CI 2.0–2.9 mm) (Figure 12). Comparison of the direct Y coordinate to the final Y coordinate showed no statistically significant difference with the mean value of the difference (Y direct-Y final) was −0.4 mm (95% CI −0.9–0.2 mm) (Figure 13). The number of the direct Y coordinates that lie within 1 mm anterior and 1 mm posterior to the final Y coordinates was 26/46 (56.5%, 95% CI = 41.6%–71.4%). Comparison of the indirect Y coordinate to the final Y coordinate showed a statistically significant difference with the mean value of the difference (Y indirect-Y final) was 0.6 mm (95% CI 0.1–1.0 mm, *p* = 0.014). The number of the indirect Y coordinates that lie within 1 mm anterior and 1 mm posterior to the final Y coordinates was 19/46 (41.3%, 95% CI = 26.5%–56.1%).

The mean value of Y coordinate of the anterior RN border was 2.3 mm lateral to MCP (SD of 1.1 mm, range 0–4.7 mm). There was no statistically significant difference between Y coordinate of the anterior RN border and Y coordinate of the final target with the mean value of the difference (Y of RN-Y final) was −0.2 mm (95% CI −0.7–0.4 mm. *p* = 0.562).

### 4.3. Z Coordinates

**Figure 14:**
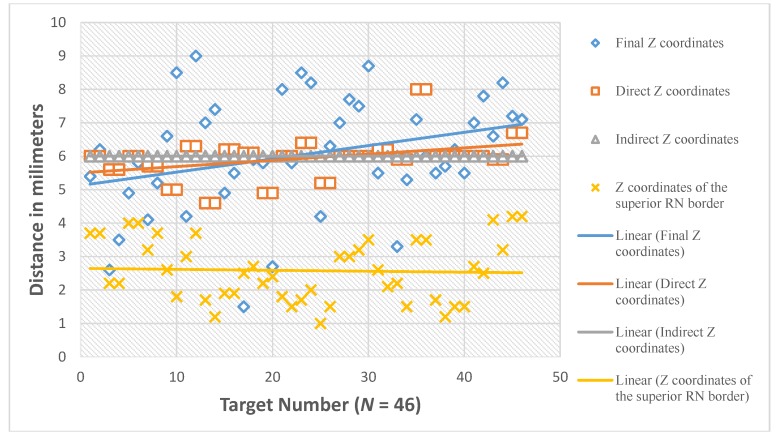
The Z coordinates in relation to the mid-commissural point (MCP).

The mean value of Z coordinate of the final active contact is 6.1 mm inferior to MCP (SD of 1.7 mm, median of 6.1, range 1.5–9.0 mm, 95% CI 5.6–6.6 mm) (Figure 14). Comparison of the direct Z coordinate to the final Z coordinate showed no statistically significant difference with the mean value of the difference (Z direct-Z final) was −0.1 mm(95% CI −0.6–0.4 mm). The number of the direct Z coordinates that lie within 1 mm superior and 1 mm inferior to the final Z coordinates is 22/46 (47.8%, 95% CI = 32.8%–62.8%). Comparison of the indirect Z coordinate to the final Z coordinate showed no statistically significant difference with the mean value of the difference (Z indirect-Z final) was −0.1 mm (95% CI −0.6–0.4 mm). The number of the indirect Z coordinates that lie within 1 mm superior and 1 mm inferior to the final Z coordinates is 22/46 (47.8%, 95% CI = 32.8%–62.8%).

**Figure 15:**
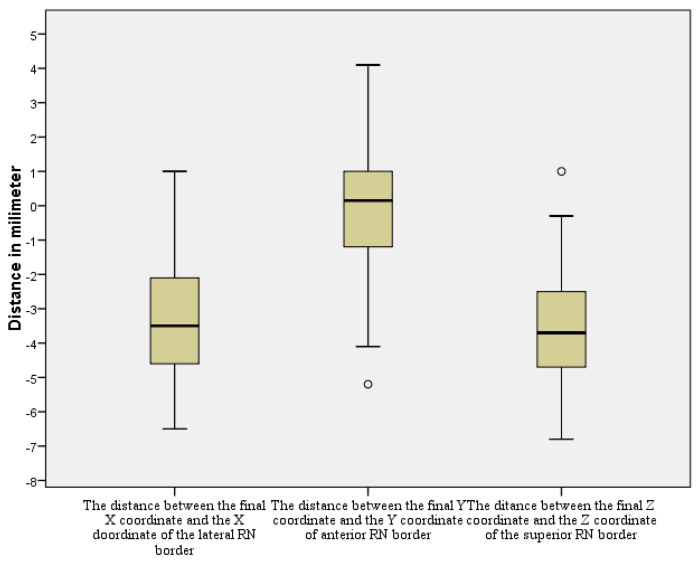
The distances between the different red nuleus (RN)borders and the final active electrode coordinates. **°**: outliers.

The mean value of Z coordinates of the superior RN border was 2.6 mm inferior to MCP (SD of 0.9 mm, range 1.0–4.2 mm). The mean distance of Z coordinates of the superior border of RN superior to the STN and Z coordinates of the final contact (Z of RN-Z final) was −3.5 mm (95% confidence interval −4.0–−2.9 mm, *p* < 0.001) (Figure 15).

### 4.4. The Euclidean Distances

The Euclidean distance (ED) between the final position of the active contact and the preoperative planned position of STN by the direct visualization method was found to be statistically significant with a mean of 2.7 mm (95% confidence interval 2.4–3.1 mm, and *p* < 0.001). The number of the direct STN targets that lie within 1 mm in any direction of the final targets is only 1/46 (2.2%, 95% CI = 0%–6.6%).

ED between the final position of the active contact and the preoperative planned position of STN by indirect method was found to be statistically significant with a mean of 2.6 mm (95% confidence interval 2.4–2.9 mm, and *p* < 0.001). The number of the indirect STN targets that lie within 1 mm in any direction of the final targets was only 2/46 (4.3%, 95% CI = 0%–10.5%).

There was no statistically significant difference in ED of the final active contact to the direct target vs. the distance of the final active contact to the indirect target with the mean difference 0.08 mm (95% confidence interval −0.3–0.4 mm, and *p* = 0.674).

## 5. Discussion

In our practice, we use both the direct and the indirect methods for the preoperative planning. However, it is not unusual to move the electrode into different coordinates of STN based on intraoperative neurophysiological findings. This may happen when our planned target does not exhibit an adequate pattern of the STN signal on MER or it turns out to be too close to the nearby structures causing undesired effects. Therefore, we may move the electrode position a few millimeters away from the originally planned one to reach a better functioning stimulation site (Figure 8). Hamani et al. [25] compared the coordinates of the different borders of STN as identified on MRI to the coordinates identified by MER of STN activity. In their results, 15 tracks (52% of the tracks) had STN-like activity outside the identified borders of STN on MRI (mostly by 1 mm) [25].

This study comes as a continuation of our previous efforts to define the optimal method to calculate the coordinates for the different DBS procedures [6,26]. We considered the final position of the active electrode contact on the postoperative MRI as the “true functional stimulation site” or the “final target”. This final target position is confirmed by the intra-operative MER and the postoperative improvement of the parkinsonian symptoms. We compared these final coordinates to the initial coordinates calculated by both the direct and indirect methods. We also studied the relations of the final functional target coordinates to the coordinates of the different borders of RN in attempt to find any new relations that can improve the preoperative planning. The reason why we chose RN is its close proximity to STN, and the fact that the borders of RN are well visualized on MRI even better than those of STN.

In our results, both the direct X and the indirect X coordinates did not show a statistically significant difference from the final X coordinate. These results confirm that X of the final functional target of STN corresponds to X of the anatomical center calculated by either of the two methods. The difference between the direct X coordinate calculation and the final X had smaller mean, variance, and narrower range, and CI than the difference between the indirect and the final X (Table 1). The mean value of X coordinate of the final active contact was 11.7 mm lateral to MCP with 95% CI = 11.2–12.2 mm. The mean distance between X coordinates of the lateral RN border and X coordinate of the final target (X of RN-X final) was 3.4 mm on the medial side, with 95% CI = 4.0–2.9 mm. Accordingly, we suggest for preoperative calculation of X coordinate to choose our X at the center of the hypointensity range representing STN on an axial MRI image, taking into consideration that most of the functional STN targets lie 11–12.5 mm lateral to MCP, and 3–4 mm lateral to the lateral RN border.

In regard to Y coordinate, comparison of the direct Y coordinate to the final Y coordinate showed no statistically significant difference. In addition, there was no statistically significant difference between the Y coordinate of the anterior RN border and the Y coordinate of the active contact. Meanwhile, a comparison of the indirect Y coordinate to the final Y coordinate showed a statistically significant difference. These results confirm that Y of the final functional target of the STN corresponds to Y of the anatomical center calculated by direct visualization on MRI at the extension of a straight line drawn at the anterior margin of the RN, as suggested by Bejjani et al. in 2000 [7]. The mean value of the Y coordinate of the final target was 2.4 mm posterior to the MCP with 95% CI = 2.0–2.9 mm. Accordingly, we suggest for the preoperative calculation of the Y coordinate to choose our Y at the center of the hypointensity range representing the STN in an axial MRI image. During the calculation, we should take into consideration that most of the functional STN targets lie 2–3 mm posterior to the MCP, and at the same Y of a straight line drawn at the anterior margin of the RN.

In regard to the Z coordinate, both the direct Z and the indirect Z coordinates did not show a statistically significant difference from the final Z coordinate. These results confirm that Z of the final functional target of STN corresponds to the Z of the anatomical center calculated by either of the two methods. The mean value of the Z coordinate of the final active contact was 6.1 mm inferior to MCP with a 95% CI = 5.6–6.6 mm. The mean distance of the Z coordinate of the superior RN border and the Z coordinate of the final contact (Z of RN-Z final) was 3.5 mm more superior, with the 95% CI = 4.0–2.9 mm. Accordingly, we suggest for the preoperative calculation of the Z coordinate to choose our Z at the center of the hypointensity representing STN on coronal MRI, taking into consideration that most of the STN functional targets lie between 5.5 and 6.6 mm inferior to MCP, and 3–4 mm inferior to the superior RN border.

The results of this study show that both the indirect and the direct methods of localization correspond largely to the final functional target in calculating X and Z coordinates, with the direct visualization being more accurate. Nevertheless, the indirect Y coordinate was significantly posterior to Y of the final optimal target. Ashkan et al. [13] calculated the indirect STN coordinates at 12 mm lateral, 2 mm posterior, and 4 mm inferior to MCP. They compared these indirect coordinates with those obtained by direct visualization. Their results showed that, on average, the directly visualized target compared to the atlas target was 1.7 mm more medial (*p* < 0.0001), 0.7 mm more anterior (*p* < 0.001) and 0.7 mm more ventral (*p* < 0.0001).

In our indirect calculations, we used the Z coordinate at 6 mm inferior to MCP. Our results showed that there was no significant difference between this Z coordinate and the final Z value. This is different from other studies that used other values of Z coordinates such as 3 [27], 4 [13,28,29,30,31], or 5 [16] mm inferior to MCP. These studies found a significant difference in the Z coordinate calculation between the indirect and the direct [16,31] targeting or between the indirect and the final target [27,30].

To our best knowledge, no studies calculated the difference in the distance between the final STN coordinates and the coordinates of the different RN borders. Andrade-Souza et al. [27] used coordinates derived from the stereotactic atlases to preoperatively plan STN targets based on the coordinates of different RN borders. They defined an X coordinate 3 mm lateral to the lateral RN border, a Y coordinate at the same Y of the anterior RN border, and a Z coordinate as 2 mm inferior to the superior RN border. They calculated the mean ± SD of the differences between the preoperative RN based calculations and the final targets; X = 1.82 ± 1.38 mm, Y = 1.62 ± 1.05 mm, and Z = 1.37 ± 0.93 mm. Houshmand et al. [16] used the same parameters used by Andrade-Souza et al. [27] to calculate STN target coordinates based on the coordinates of different RN borders. They calculated the distances between different RN borders and the STN anatomical center seen on 3T MRI. They calculated the mean ± SD of the differences between the preoperative RN based calculation and the final targets; X = 0.67 ± 0.45 mm, Y = 0.77 ± 0.54 mm, and Z = 0.56 ± 0.40 mm. Both of those studies found that the RN base targeting was closer to the optimal target, than the direct or the indirect calculations. Starr et al. [29] considered the center of the DBS electrode array in the postoperative MRI as the final target, and they calculated the mean distance between its coordinates and X and Y coordinates of the center of RN (X = 6.5 mm lateral, Y = 3.5 mm anterior).

Despite the great similarities of the final coordinates to that of the preoperatively planned direct and indirect coordinates, the Euclidean distances between the final targets and both the direct and indirect targets were found to have statistically significantly differences. This in addition to the wide range of values of the different coordinates of the final targets in relation to MCP (X = 8.2–16.0, Y = 0.0–6.2, Z = 1.5–9.0) exclude the possibility of depending on the preoperative planning as the sole method of targeting STN. We believe that the intra-operative physiological and clinical confirmation of the target is crucial in the final position confirmation. Still, initial anatomical and radiological planning is also essential in target selection. Accurate preoperative planning would decrease the intra-operative time needed for MER, and the number of microelectrodes tracks needed to reach the target, and subsequently prevent additional complication. This fact is supported by our experience, as our average number of MER tracks was 1.4 tracks.

## 6. Conclusions

The functional target of STN corresponds to the anatomical center of STN as seen in the three orthogonal planes of MRI images. During the preoperative calculation of the STN target, we prefer using the direct method, and taking into consideration that most of the functional targets are located: (1) 11–12.5 mm lateral to MCP, and 3–4 mm lateral to the lateral RN border; (2) 2–3 mm posterior to MCP, and at the same Y of a straight line drawn at the anterior margin of the red nuclei; (3) 5.5–6.6 mm inferior to MCP, and 3–4 mm inferior to the superior RN border. It seems that the preoperative anatomical/radiological planning cannot be used as the sole method of targeting the STN, intra-operative physiological and clinical confirmation is crucial in the final position confirmation.

## Figures and Tables

**Table 1:** The differences between the direct and indirect coordinates of STN from the final active electrode coordinates.

Method	Directions	Mean ± SD (mm)	Variance (mm)	Range (mm)	95% CI (mm)	*p*
Direct	X	−0.2 ± 1.5	2.2	−2.9–3.0	−0.7–0.2	0.271
	Y	−0.4 ± 1.9	3.6	−4.9–6.5	−0.9–0.2	0.205
	Z	−0.1 ± 1.7	3.0	−3.5–4.6	−0.6–0.4	0.650
	ED	2.7 ± 1.2	1.5	0.4–6.8	2.4–3.1	<0.001 *
Indirect	X	0.3 ± 1.5	2.3	−4.0–3.8	−0.2–0.8	0.186
	Y	0.6 ± 1.5	2.3	−3.2–3.0	0.1–1.0	0.014 *
	Z	−0.1 ± 1.7	2.9	−3.0–4.5	−0.6–0.4	0.810
	ED	2.6 ± 0.9	0.8	0.9–4.5	2.4–2.9	<0.001 *

Total = 46 targets, SD: standard deviation, CI: Confidence interval, *p*: *p*-value of one sample *t*-test, ED: Euclidean distance, * Significant: Differences are direct/indirect—final: Positive-X = lateral to the final X, Negative-X = medial to the final X; Positive-Y = posterior to the final Y, Negative-Y = anterior to the final Y; Positive-Z = inferior to the final Z, Negative-Z = superior to the final Z.

**Table 2:** Numbers and percentages of the coordinates of the direct, indirect and RN borders within ±1.0 mm of the final targets.

Method	Coordinates	*N* (%)	95% CI
Direct	X	24 (52.2%)	37.2%–67.2%
	Y	26 (56.5%)	41.6%–71.4%
	Z	22 (47.8%)	32.8%–62.8%
	ED	1 (2.2%)	0.0%–6.6%
Indirect	X	23 (50.0%)	35.0%–65.0%
	Y	19 (41.3%)	26.5%–56.1%
	Z	22 (47.8%)	32.8%–62.8%
	ED	2 (4.3%)	0.0%–10.5%
Y coordinate of the anterior RN border		22 (47.8%)	32.8%–62.8%

Total = 46 targets, CI: Confidence interval, ED: Euclidean distance, RN: red nucleus.

**Table 3:** The distances between the different RN borders and the final active electrode coordinates.

Coordinates	Mean ± SD (mm)	Variance (mm)	Range (mm)	95% CI (mm)	*p*
X of the lateral RN border	−3.4 ± 1.8	3.3	−6.5–1.0	−4.0–−2.9	<0.001 *
Y of the anterior RN border	−0.2 ± 1.9	3.7	−5.2–4.1	−0.7–0.4	0.562
Z of the superior RN border	−3.5 ± 1.8	3.3	−6.8–1.0	−4.0–−2.9	<0.001 *

Total = 46 targets, SD: standard deviation, CI: Confidence interval, *p*: *p*-value of one sample *t*-test, * Significant: Differences are direct/indirect—final: Positive-X = lateral to the final X, Negative-X = medial to the final X; Positive-Y = posterior to the final Y, Negative-Y = anterior to the final Y; Positive-Z = inferior to the final Z, Negative-Z = superior to the final Z.

## References

[1] Sukul V.V., Slavin K.V. (2014). Deep brain and motor cortex stimulation. Curr. Pain Headache Rep..

[2] Parent A., Hazrati L.N. (1995). Functional anatomy of the basal ganglia. II. The place of subthalamic nucleus and external pallidum in basal ganglia circuitry. Brain Res. Brain Res. Rev..

[3] Hamani C., Saint-Cyr J.A., Fraser J., Kaplitt M., Lozano A.M. (2004). The subthalamic nucleus in the context of movement disorders. Brain.

[4] Rodriguez-Oroz M.C., Rodriguez M., Guridi J., Mewes K., Chockkman V., Vitek J., DeLong M.R., Obeso J.A. (2001). The subthalamic nucleus in Parkinson’s disease: Somatotopic organization and physiological characteristics. Brain.

[5] Levy R., Hutchison W.D., Lozano A.M., Dostrovsky J.O. (2000). High-frequency synchronization of neuronal activity in the subthalamic nucleus of parkinsonian patients with limb tremor. J. Neurosci..

[6] Slavin K.V., Anderson G.J., Burchiel K.J. (1999). Comparison of three techniques for calculation of target coordinates in functional stereotactic procedures. Stereotact. Funct. Neurosurg..

[7] Bejjani B.P., Dormont D., Pidoux B., Yelnik J., Damier P., Arnulf I., Bonnet A.M., Marsault C., Agid Y., Philippon J. (2000). Bilateral subthalamic stimulation for Parkinson’s disease by using three-dimensional stereotactic magnetic resonance imaging and electrophysiological guidance. J. Neurosurg..

[8] Slavin K.V., Thulborn K.R., Wess C., Nersesyan H. (2006). Direct visualization of the human subthalamic nucleus with 3T MR imaging. Am. J. Neuroradiol..

[9] Hariz M.I., Krack P., Melvill R., Jorgensen J.V., Hamel W., Hirabayashi H., Lenders M., Wesslen N., Tengvar M., Yousry T.A. (2003). A quick and universal method for stereotactic visualization of the subthalamic nucleus before and after implantation of deep brain stimulation electrodes. Stereotact. Funct. Neurosurg..

[10] Dormont D., Ricciardi K.G., Tande D., Parain K., Menuel C., Galanaud D., Navarro S., Cornu P., Agid Y., Yelnik J. (2004). Is the subthalamic nucleus hypointense on T2-weighted images? A correlation study using MR imaging and stereotactic atlas data. Am. J. Neuroradiol..

[11] Wippold F.J., Brown D.C., Broderick D.F., Burns J., Corey A.S., Deshmukh T.K., Douglas A.C., Holloway K., Jagadeesan B.D., Jurgens J.S. (2015). ACR appropriateness criteria dementia and movement disorders. J. Am. Coll. Radiol..

[12] Chandran A.S., Bynevelt M., Lind C.R. (2016). Magnetic resonance imaging of the subthalamic nucleus for deep brain stimulation. J. Neurosurg..

[13] Ashkan K., Blomstedt P., Zrinzo L., Tisch S., Yousry T., Limousin-Dowsey P., Hariz M.I. (2007). Variability of the subthalamic nucleus: The case for direct MRI guided targeting. Br. J. Neurosurg..

[14] Littlechild P., Varma T.R., Eldridge P.R., Fox S., Forster A., Fletcher N., Steiger M., Byrne P., Tyler K., Flintham S. (2003). Variability in position of the subthalamic nucleus targeted by magnetic resonance imaging and microelectrode recordings as compared to atlas co-ordinates. Stereotact. Funct. Neurosurg..

[15] Patel N.K., Khan S., Gill S.S. (2008). Comparison of atlas- and magnetic-resonance-imaging-based stereotactic targeting of the subthalamic nucleus in the surgical treatment of Parkinson’s disease. Stereotact. Funct. Neurosurg..

[16] Houshmand L., Cummings K.S., Chou K.L., Patil P.G. (2014). Evaluating indirect subthalamic nucleus targeting with validated 3-Tesla magnetic resonance imaging. Stereotact. Funct. Neurosurg..

[17] Caire F., Ouchchane L., Coste J., Gabrillargues J., Derost P., Ulla M., Durif F., Lemaire J.J. (2009). Subthalamic nucleus location: Relationships between stereotactic AC-PC-based diagrams and MRI anatomy-based contours. Stereotact. Funct. Neurosurg..

[18] Talairach J., Tournoux P. (1988). Co-Planar Stereotaxic Atlas of the Human Brain: 3-Dimensional Proportional System: An Approach to Cerebral Imaging.

[19] Starr P.A., Vitek J.L., DeLong M., Bakay R.A. (1999). Magnetic resonance imaging-based stereotactic localization of the globus pallidus and subthalamic nucleus. Neurosurgery.

[20] Niemann K., Mennicken V.R., Jeanmonod D., Morel A. (2000). The Morel stereotactic atlas of the human thalamus: Atlas-to-MR registration of internally consistent canonical model. Neuroimage.

[21] Schaltenbrand G., Wharen W. (1977). Atlas for Stereotaxy of the Human Brain.

[22] Hutchison W.D., Allan R.J., Opitz H., Levy R., Dostrovsky J.O., Lang A.E., Lozano A.M. (1998). Neurophysiological identification of the subthalamic nucleus in surgery for Parkinson’s disease. Ann. Neurol..

[23] Saint-Cyr J.A., Hoque T., Pereira L.C., Dostrovsky J.O., Hutchison W.D., Mikulis D.J., Abosch A., Sime E., Lang A.E., Lozano A.M. (2002). Localization of clinically effective stimulating electrodes in the human subthalamic nucleus on magnetic resonance imaging. J. Neurosurg..

[24] Vergani F., Landi A., Antonini A., Parolin M., Cilia R., Grimaldi M., Ferrarese C., Gaini S.M., Sganzerla E.P. (2007). Anatomical identification of active contacts in subthalamic deep brain stimulation. Surg. Neurol..

[25] Hamani C., Richter E.O., Andrade-Souza Y., Hutchison W., Saint-Cyr J.A., Lozano A.M. (2005). Correspondence of microelectrode mapping with magnetic resonance imaging for subthalamic nucleus procedures. Surg. Neurol..

[26] Colpan M.E., Slavin K.V. (2010). Subthalamic and red nucleus volumes in patients with Parkinson’s disease: Do they change with disease progression?. Parkinsonism Relat. Disord..

[27] Andrade-Souza Y.M., Schwalb J.M., Hamani C., Eltahawy H., Hoque T., Saint-Cyr J., Lozano A.M. (2005). Comparison of three methods of targeting the subthalamic nucleus for chronic stimulation in Parkinson’s disease. Neurosurgery.

[28] Benabid A.L., Pollack P., Benazzouz A. (1998). Grenoble guidelines for deep brain stimulation. First European Symposium on Stimulation in Parkinson Disease.

[29] Starr P.A., Christine C.W., Theodosopoulos P.V., Lindsey N., Byrd D., Mosley A., Marks W.J. (2002). Implantation of deep brain stimulators into the subthalamic nucleus: Technical approach and magnetic resonance imaging-verified lead locations. J. Neurosurg..

[30] Hamid N.A., Mitchell R.D., Mocroft P., Westby G.W., Milner J., Pall H. (2005). Targeting the subthalamic nucleus for deep brain stimulation: Technical approach and fusion of pre- and postoperative MR images to define accuracy of lead placement. J. Neurol. Neurosurg. Psychiatry.

[31] Savas A., Bozkurt M., Akbostanci C. (2013). A comparison between stereotactic targeting methods of the subthalamic nucleus in cases with Parkinson’s disease. Acta Neurochir. Suppl..

